# Buprenorphine is a weak dopamine releaser relative to heroin, but its pretreatment attenuates heroin‐evoked dopamine release in rats

**DOI:** 10.1002/npr2.12139

**Published:** 2020-09-15

**Authors:** Dominic P. Isaacs, Ryan P. Leman, Thomas J. Everett, Hendrick Lopez‐Beltran, Lindsey R. Hamilton, Erik B. Oleson

**Affiliations:** ^1^ Psychology Department University of Colorado Denver Colorado USA; ^2^ Department of Bioengineering University of Colorado Denver Colorado USA

**Keywords:** addiction, agonist therapy, dopamine, opioid, substitution therapy, voltammetry

## Abstract

**Aims:**

The United States of America is currently in an opioid epidemic. Heroin remains the most lethal opioid option with its death rate increasing by over 500% in the last decade. The rewarding and reinforcing effects of heroin are thought to be mediated by its ability to increase dopamine concentration in the nucleus accumbens shell. By activating Gi/o‐coupled μ‐opioid receptors, opioids are thought to indirectly excite midbrain dopamine neurons by removing an inhibitory GABAergic tone. The partial μ‐opioid receptor agonist buprenorphine is a substitution‐based therapy for heroin dependence that is thought to produce a steady‐state level of μ‐opioid receptor activation. But it remains unclear how buprenorphine alters dopamine release relative to heroin and how buprenorphine alters the dopamine‐releasing effects of heroin. Because buprenorphine is a partial agonist at the μ‐opioid receptor and heroin is a full agonist, we predicted that buprenorphine would function as a weak dopamine releaser relative to heroin, while functioning as a competitive antagonist if administered in advance of heroin.

**Methods:**

We performed fast‐scan cyclic voltammetry in awake and behaving rats to measure how heroin, buprenorphine HCl, and their combination affect transient dopamine release events in the nucleus accumbens shell. We also performed a complimentary pharmacokinetic analysis comparing opioid plasma levels at time points correlated to our neurochemical findings.

**Results:**

Both buprenorphine and heroin produced changes in the frequency of transient dopamine release events, although the effect of buprenorphine was weak and only observed at a low dose. In comparison with vehicle, the frequency of dopamine release events maximally increased by ~25% following buprenorphine treatment and by ~60% following heroin treatment. Distinct neuropharmacological effects were observed in the high‐dose range. The frequency of dopamine release events increased linearly with heroin dose but biphasically with buprenorphine dose. We also found that buprenorphine pretreatment occluded the dopamine‐releasing effects of heroin, but plasma levels of buprenorphine had returned to baseline at this time point.

**Conclusion:**

These findings support the notion that low‐dose buprenorphine is a weak dopamine releaser relative to heroin and that buprenorphine pretreatment can block the dopamine‐releasing effects of heroin. The finding that high‐dose buprenorphine fails to increase dopamine release might explain its relatively low abuse potential among opioid‐dependent populations. Because high‐dose buprenorphine decreased dopamine release before occluding heroin‐evoked dopamine release, and buprenorphine was no longer detected in plasma, we conclude that the mechanisms through which buprenorphine blocks heroin‐evoked dopamine release involve multifaceted pharmacokinetic and pharmacodynamic interactions.

## Abstract 2

Buprenorphine functions as a weak dopamine releaser relative to heroin but its pretreatment occludes the dopamine‐releasing effects of heroin. These data may help explain why buprenorphine poses minimal risk for abuse yet functions as an effective substitution therapy for opioid dependence.

## Graphical abstract

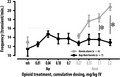

## 1 INTRODUCTION

The United States of America is currently in an opioid epidemic that is producing over 100 deaths per day. Fentanyl and heroin are the most lethal opioid options, with their respective death rates increasing by 520% and 533% between 2009 and 2016.^1^ The rewarding and reinforcing effects of opioids are principally mediated through their action at the μ‐opioid receptor.^2^ Following the development of opioid dependence, Subutex^®^ and Suboxone^®^ are commonly prescribed pharmacotherapies for its treatment.^3^ Subutex^®^ is buprenorphine; Suboxone^®^ is a 4:1 ratio of buprenorphine to naloxone. The partial μ‐opioid receptor agonist buprenorphine is thought to act as a substitution therapy in opioid dependence; the μ‐opioid receptor antagonist naloxone is primarily added to Suboxone^®^ in order to prevent intravenous (IV) buprenorphine abuse.^4^, ^5^

Heroin produces its rewarding and reinforcing effects by increasing dopamine concentration in the mesolimbic dopamine pathway,^6^, ^7^, ^8^, ^9^, but also see Ettenberg et al, 1982.^10^ By activating μ‐opioid receptors on midbrain GABA neurons, opioids are thought to disinhibit dopamine neurons in the ventral tegmental area (VTA).^6^ In the awake and behaving animal, dopamine neurons in the VTA—the origin of the mesolimbic pathway—fire in two distinct patterns^11^ when at rest, dopamine neurons fire in a slow (2‐5 Hz) pacemaker pattern that contributes to a steady‐state dopamine tone. This low‐concentration tone is thought to activate high‐affinity dopamine receptors (eg, D2) in terminal regions of the mesolimbic pathway.^11^ Dopamine neurons also fire in phasic bursts (>20 Hz) under a variety of conditions, including when animals are presented with rewarding and motivationally salient stimuli. These phasic bursts of neural activity contribute to transient dopamine release events that are sufficient in concentration to occupy low‐affinity dopamine D1 receptors.^11^, ^12^ Because only D1‐expressing neurons in the nucleus accumbens (NAc)—the primary terminal field of the mesolimbic pathway—undergo dendritic plasticity following repeated drug exposure,^13^ studying how opioids alter high‐concentration dopamine transients may be particularly important for the neurobiology of addiction.

By investigating the effects of opioids on dopamine transients, we sought to build upon the existing microdialysis, electrochemistry, and electrophysiology literature. Previous publications show that heroin increases dopamine concentration in the NAc shell^6^, ^7^, ^8^, ^9^ and that buprenorphine pretreatment blocks several behavioral and neurochemical effects of heroin^14^, ^15^—including its ability to increases accumbal dopamine concentration. This latter finding is also supported by the behavioral pharmacology literature, which shows that buprenorphine can function as a competitive antagonist in the presence of other opioid ligands.^16^, ^17^ Because buprenorphine is a partial agonist at the μ‐opioid receptor, when administered in advance it should compete with and therefore obstructs the full agonist 6‐acetylmorphine (ie, the active metabolite of heroin) from evoking dopamine release. Therefore, we predicted that buprenorphine would function as a relatively weak dopamine releaser in the absence of heroin, but its pretreatment would block the dopamine‐releasing effects of heroin.

**FIGURE 1:**
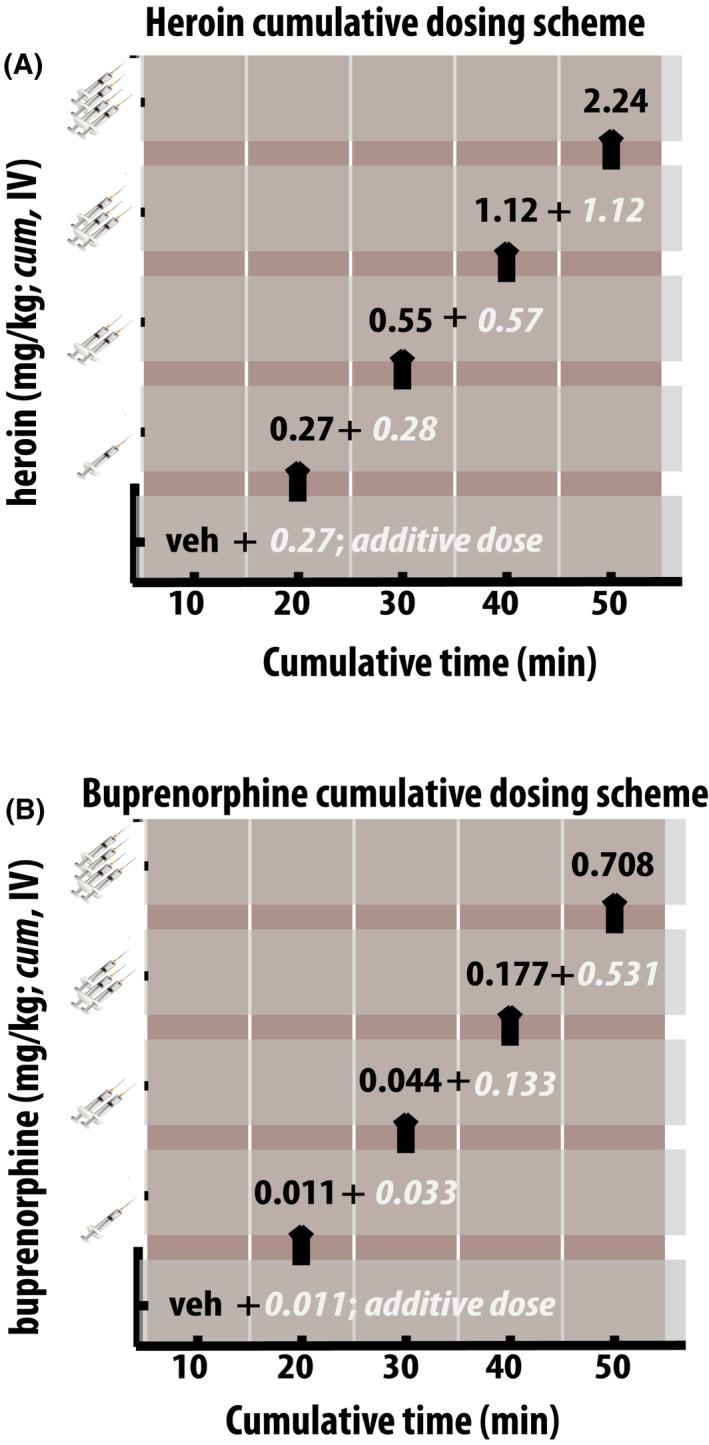
Cumulative‐dosing scheme for heroin (A) and buprenorphine (B) conditions

To test these predictions, we used fast‐scan cyclic voltammetry (FSCV) to measure drug‐induced changes in dopamine release in the awake and freely moving rat. While monitoring transient release events in the NAc shell, we treated rats with ascending intravenous (IV) doses of either: heroin alone or buprenorphine followed by heroin (Figure 1). To further investigate how opioid plasma dynamics relate to opioid‐evoked changes in dopamine transients, we performed a complimentary pharmacokinetic (PK) study. We selected our dose ranges from a clinical investigation^18^ that studied how ascending doses of IV buprenorphine and heroin alter the subjective drug experience—observations that are impossible to assess in the rat. Our FSCV findings are discussed within the context of these previously reported dose‐dependent subjective effects.

## 2 METHODS

### 2.1 Subjects and surgery

Catheterized male Long‐Evans rats, supplied by Charles River, were singly housed under a 12:12‐h light‐dark cycle with a 10 AM to 10 PM active period (dark phase). All experiments were conducted in the active phase. Rats (275‐325 g at the time of surgery) were placed under isoflurane anesthesia (5% induction, 2% maintained) for surgery conducted in a stereotaxic apparatus. A guide cannula that mates with a micromanipulator was implanted to be aimed at the NAc shell (+1.7AP, +0.8ML relative to bregma). The shell region of the NAc was targeted because the initial effects of drugs on dopamine concentration are most pronounced in this region.^19^, ^20^ In addition, a Ag/AgCl reference electrode was implanted on the contralateral side of the brain. Rats were given three days to recover before experiments were conducted.

### 2.2 FSCV

Voltammetric recordings were conducted by lowering a glass‐encased carbon fiber microelectrode using a micromanipulator that fits inside the implanted guide cannula. An initial waveform (−0.4 to 1.3 V; 400 V/s) was applied which allowed for the detection of dopamine from cyclic voltammograms taken every 100 ms. To increase electrode sensitivity, the waveform was first applied at 60 Hz for ~30 minutes but reduced to 10 Hz before experimentation. To extract the dopamine component, principle component regression was applied to the raw voltammetric data.^21^ Specifically, we used recording‐specific training sets (n = 7/analyte; dopamine and pH) to produce pH and background subtracted (10 consecutive scans) dopamine concentration files for transient analysis. To quantify dopamine concentration, calibration factors were predetermined using linear regression.^22^ Lab‐specific coefficients and additional detail into our calibration approach are available in the supplemental material of a previous publication.^23^ The resulting calibration factors allow us to calculate molar concentrations of dopamine in a session‐specific manner using observed total background current. At the end of the experiment, animals were killed with CO_2_ and electrolytic lesions were performed to confirm electrode placement. Brains were extracted and frozen in −25°C 2‐methylbutane and then stored at −80°C until they were coronally sectioned at 50 μm using a cryostat. Slices were dehydrated with baths of increasing ethanol concentration, preserved with histoclear, and mounted for observation of lesion placement.

### 2.3 Transient analysis

**FIGURE 2:**
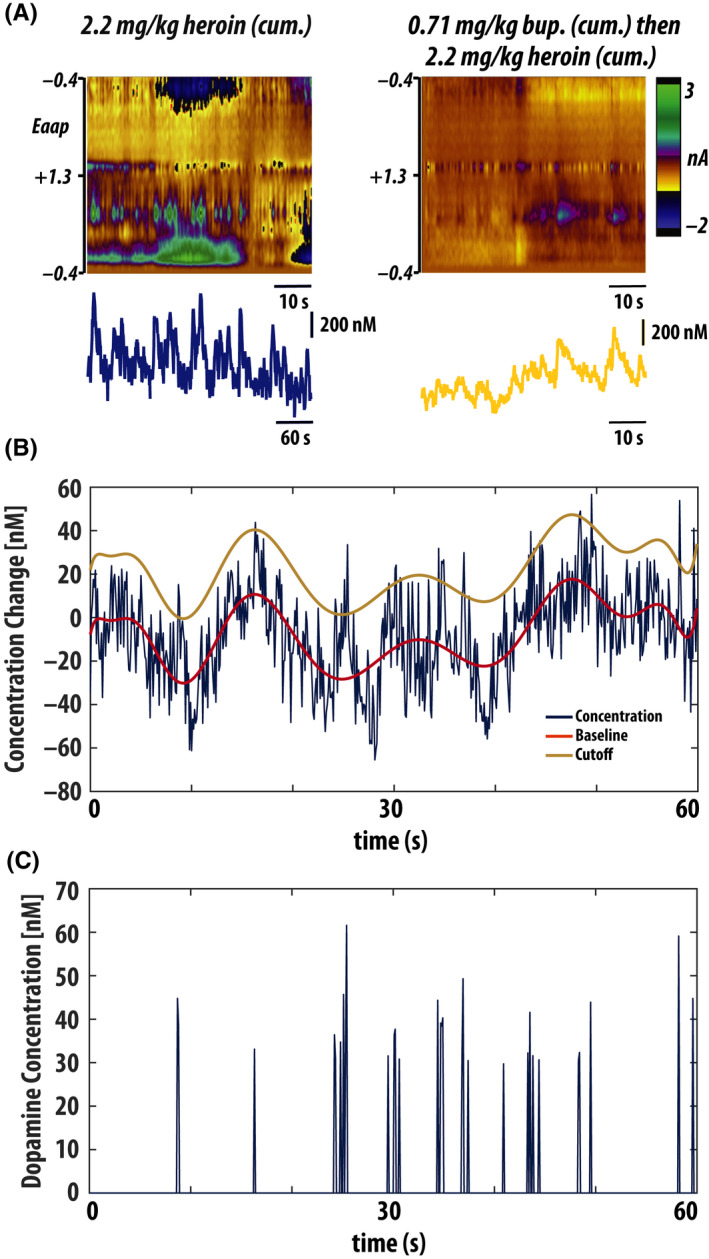
In vivo voltammetry and signal processing. A, Representative color plots (top) and dopamine concentration traces (bottom) show the effects of heroin‐alone (left) and buprenorphine + heroin (right) on accumbal dopamine release. In the color plots, voltammetric current (*z*‐axis) is plotted against applied scan potential (Eapp; *y*‐axis) and time (*x*‐axis). B, To quantify the frequency and amplitude of drug‐induced changes in dopamine release, two polynomic lines are first fitted to each set of dopamine concentration data. C, Only dopamine release events with concentrations that were 1.5 standard deviations above the lower “baseline” were included in subsequent dose assessments

As previously described,^24^ for every 60 seconds recording, a peak‐threshold (“cutoff”) polynomic line was fitted to each set of dopamine concentration data using the following equation (Figure 2):

$$p\left(x\right)=p_{1}x^{2}{+p}_{2}x^{2}{+p}_{3}.$$

The coefficients (*p*_1_, *p*_2_, p_3_) with the largest *R* ^2^ value were assigned for each individual 60 seconds dopamine concentration file. The degree of the polynomial was determined by finding the lowest Akaike information criterion (AIC) score with the following equation:

$$\text{AIC}=n*\log\left(\frac{\text{RSS}}{n}\right)+2K.$$

A second fitted line was generated to establish a relative zero (“baseline”) using the following equation:

$$p\left(x\right)=p_{1}x^{n}+p_{2}x^{n‐1}+\cdots p_{n}x+p_{n}+1.$$

To reduce type 1 error, only peaks above 1.5 standard deviation from the “baseline” that were greater than 0.5 seconds apart were analyzed. If multiple peaks occurred within the 0.5 seconds period, the highest concentration event was reported.

### 2.4 Pharmacology

Heroin and buprenorphine were prepared in sterile saline and briefly sonicated. In all experiments, opioids were administered intravenously using a cumulative‐dosing approach. For the heroin‐only condition, we administered vehicle (ie sterile saline) and then 0.27, 0.55, 1.12, and 2.24 heroin (mg/kg IV). Cumulative doses were administered every 10 minutes as illustrated in Figure 1A.

In the buprenorphine‐heroin condition, we administered vehicle (ie sterile saline) and then ascending doses of buprenorphine: 0.011, 0.044, 0.177, 0.708 (Figure 1B). Continuing in 10‐minutes intervals, we then administered the same doses of heroin used in the heroin‐only condition. Buprenorphine HCl was purchased from Sigma‐Aldrich, and heroin was provided by the NIDA drug supply program. Doses of buprenorphine were calculated as the weight of the HCl salt.

### 2.5 Comparative dosing calculations

In the present study, we took advantage of a unique clinical investigation in which human participants were given ascending doses of intravenous buprenorphine and heroin—in addition to several other opioids, and then asked to subjectively rate the resulting experience/affect (eg “I feel good”).^18^ In addition, the propensity to self‐administer each dose was assessed. To convert these previously compared opioid doses in the human literature for use in the rat, we applied the following allometric scaling equation.^25^

$$\mathrm{Human}\mathrm{dose}\left(\frac{\mathrm{mg}}{\mathrm{kg}}\right)=\mathrm{animal}\mathrm{dose}\left(\frac{\mathrm{mg}}{\mathrm{kg}}\right)\times \left(\frac{\mathrm{RatKm}}{\mathrm{HumanKm}}\right)$$

The Km used for human = 37; the Km used for rat = 6.2.^25^

### 2.6 Pharmacokinetic analysis

To determine circulating concentrations of buprenorphine, heroin, and its metabolites, we performed a pharmacokinetic analysis. Long‐Evans rats were either treated with cumulative heroin doses only or pretreated with cumulative buprenorphine doses followed by cumulative heroin doses. The cumulative‐dosing scheme for heroin and buprenorphine ([0.2, 0.4, 1.1, 2.2] mg/kg and [0.01, 0.04, 0.18, 0.7] mg/kg, respectively) was identical to the voltammetry experiment dosing scheme previously described. 0.5 mL of whole blood was taken from animals via femoral vein catheter and placed in KDTA‐coated blood collection tubes at regular time intervals postdrug infusion. For the heroin‐only group (Figure 7A), blood was taken every 10 minutes. For the buprenorphine‐pretreated group, blood was taken after cumulative dosing of buprenorphine and also after heroin cumulative dosing. Blood samples were centrifuged at 750 *g* for 12 minutes to separate components. Separated plasma was collected and sent to the Pharmacology core at Colorado State University for combined liquid chromatography/mass spectroscopy (LC‐MS), which consists of a Shimadzu HPLC machine with a Waters Sunfire C18 5 µm 4.6 × 150 mm elution column (Part No. 186002557) coupled to a 3200 Q‐TRAP triple quadrupole mass spectrometer (Applied Biosystems, Inc).

#### 2.6.1 Generation of standard curves utilized in LC‐MS

A 0.1 mg/mL stock solution of buprenorphine, morphine, M3G, and 6‐acetylmorphine (6‐AM) in 50/50 ACN/Milli‐Q was prepared. Standard curves were created using the following concentrations (10, 25, 50, 100, 250, 500, 1000, 2500, 5000, 10 000, 25 000, and 50 000) ng/mL buprenorphine, morphine, M3G, and 6‐AM in 50/50 ACN/Milli‐Q. A 100 ng/mL solution of fentanyl in 50/50 ACN/Milli‐Q was prepared as an internal standard. Next, 5 µL of each standard was added to fresh 1.5‐mL microcentrifuge tubes, along with 5 µL of 500 ng/mL fentanyl solution. 9 Quality control samples (3 × 10, 3 × 100, and 3 × 1000) ng/mL were prepared in the same manner. 50 µL of blank Rat plasma and 50 µL of acetonitrile with 0.1% formic acid were added to each tube and vortexed for 5 minutes. The tubes were then centrifuged for 15 minutes at 20 000 *g* and transferred to an autosampler with inserts.

#### 2.6.2 Experimental sample preparation

Five microlitre of 50/50 ACN/Milli‐Q, 5 µL of 100 ng/mL fentanyl standard, 50 µL of unknown experimental rat plasma sample, and 50 µL of ACN with 0.1% formic acid were added to fresh 1.5‐mL microcentrifuge tubes. The tubes were vortexed for 5 minutes, centrifuged for 15 minutes at 20 000 *g*, and transferred to an autosampler vials with inserts.

### 2.7 Description of the statistical analysis

Because parametric statistical tests such as ANOVA assume that experimental samples have equal variance with data that are normally distributed, we first performed tests of normality and equal variance. If data were determined to normally distribute and show equal variance, we assessed for significant differences in the data using parametric statistics (eg, ANOVA). In contrast, if data were determined to not fit these criteria, we proceeded to assess for significant differences in the data using nonparametric statistics. Final statistical analyses were performed using SigmaPlot 11.

## 3 RESULTS

### 3.1 Determination of animal equivalent heroin dosage for rat

To assess the effects of heroin, buprenorphine, and their interaction on transient dopamine release events, we treated rats with cumulative ascending IV doses of either heroin‐alone (n = 6) or buprenorphine followed by heroin (n = 7) while concurrently performing FSCV in the freely moving rat. Doses were adapted from a clinical study that asked opioid‐dependent humans to compare the subjective effects of ascending doses of buprenorphine (0.125, 0.5, 2, and 8 mg/70 kg IV) and heroin (3.125, 6.25, 12.5, and 25 mg/70 kg IV). Our calculated rat animal equivalent doses (AEDs) were (0.01, 0.04, 0.18, and 0.7 mg/kg IV) for buprenorphine and (0.27, 0.55, 1.1, and 2.2 mg/kg IV) for heroin.^25^ We have repeatedly demonstrated that repeated saline injections do not influence dopamine release events^24^, ^26^ and confirmed^24^ accounts from the historical literature^27^, ^28^ that electrode sensitivity remains stable over the course of our in vivo recording sessions.

### 3.2 Buprenorphine is a weak dopamine releaser vs. heroin and its pretreatment blocked heroin‐evoked dopamine release

**FIGURE 3:**
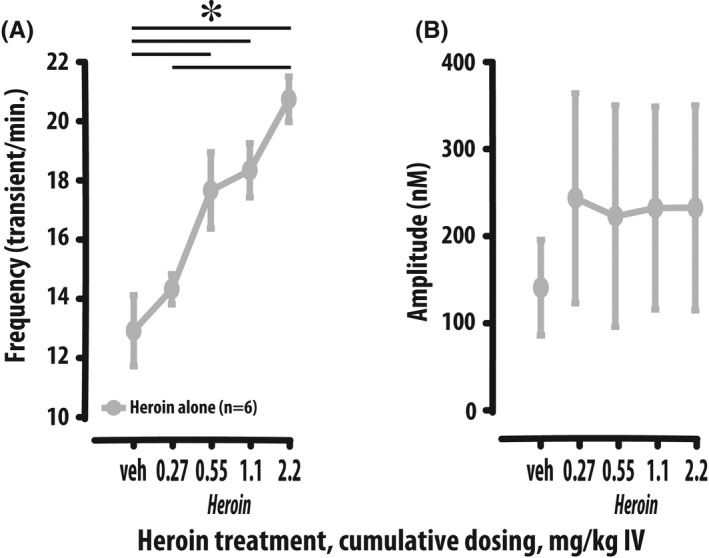
Heroin increased the frequency of dopamine release events in a linear dose‐dependent manner. A, Heroin increased the frequency of dopamine release events in a linear, dose‐dependent manner. B, The effect of heroin dose on the amplitude of dopamine release events was less resolved and did not occur in a dose‐dependent manner. Horizontal bars indicate significance between two specific treatments. *P < .05

**FIGURE 4:**
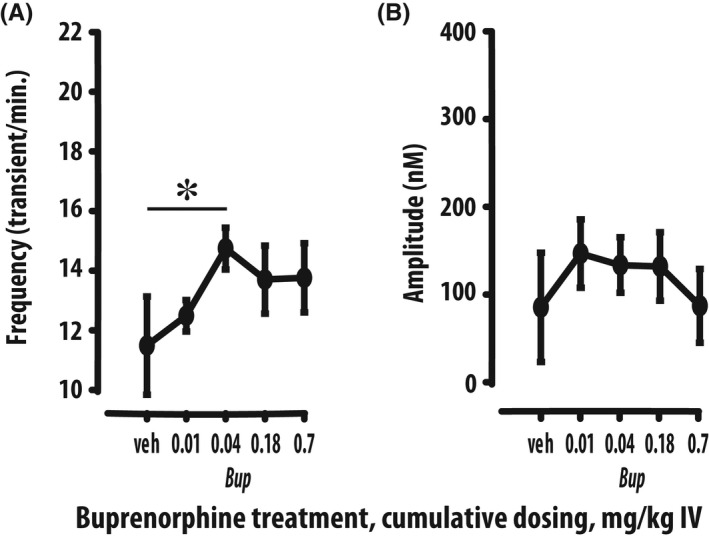
Buprenorphine increased the frequency of dopamine release events in a bell‐shaped dose‐dependent manner. A, Buprenorphine increased the frequency of dopamine release events in a bell‐shaped dose‐dependent manner. Maximal release was observed at the 0.04 mg/kg IV dose and then decreased to an insignificant level in the 0.18‐0.7 mg/kg IV range. B, The effect of buprenorphine dose on the amplitude of dopamine release events was less resolved and insignificant vs vehicle. Horizontal bars indicate significance between two specific treatments. *P < .05

**FIGURE 5:**
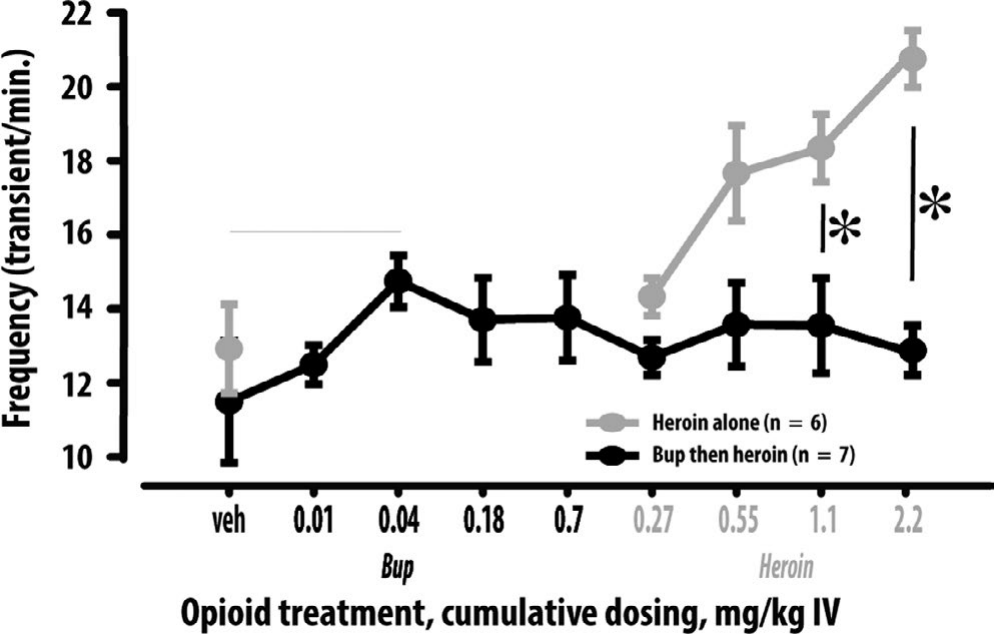
Buprenorphine pretreatment attenuates heroin‐evoked dopamine release. Heroin only (grey) increased the frequency of dopamine release events in a lawful, dose‐dependent manner. Buprenorphine (black, 1st four dosing increments after vehicle) increased the frequency of dopamine release events in a bell‐shaped dose‐dependent manner. In comparison with heroin‐alone rats, buprenorphine pretreatment also attenuated the ability of heroin to evoke dopamine release (black, last four dosing increments). Vertical bars indicate between‐group comparison. *P < .05

**FIGURE 6:**
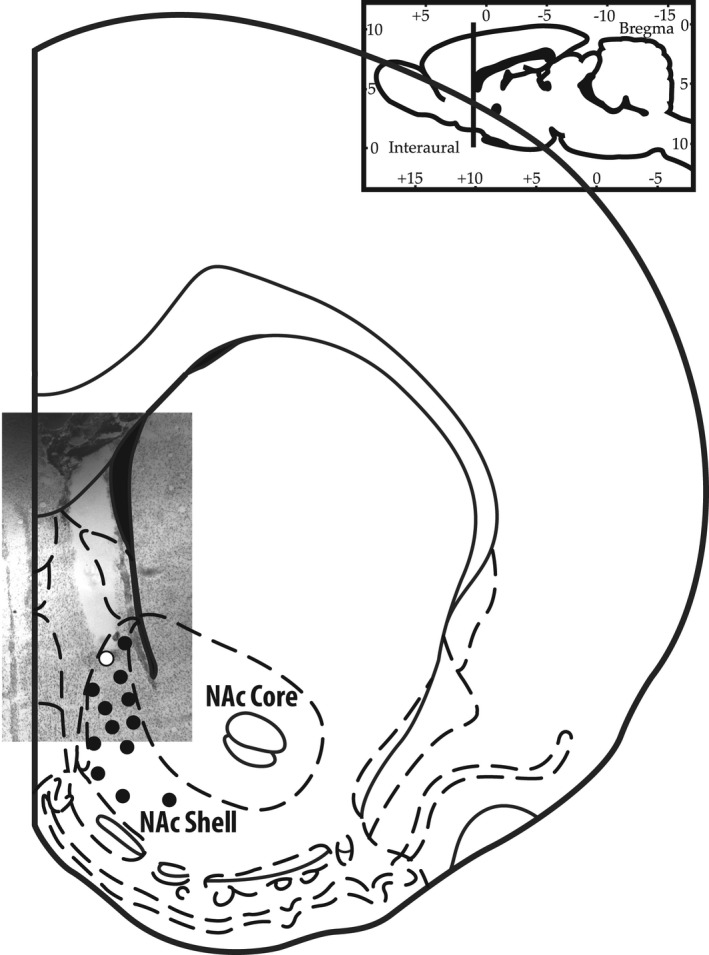
Histological reconstruction of working electrode placements during FSCV measurements. Lighter red dot corresponds to representative histology that showing lesion

As illustrated in Figures 3 and 4, buprenorphine and heroin increased the frequency of dopamine release events by ~25% and ~60%, respectively. Amplitude did not change in a significant or lawful dose‐dependent manner; however, it is possible that relatively high variability in this measure might be masking dose‐dependent effects. Frequency data first passed tests of normality (Shapiro‐Wilk) and equal variance; thus, we proceeded with parametric analyses. Criterion for significant was predetermined to be *P* < .05. All doses were tested cumulatively, with 10 minutes elapsing between each IV treatment. A one‐way repeated‐measures ANOVA revealed that heroin *F*_4,29_ = 14.12, *P* < .05 (Figure 3) and buprenorphine *F*_4,34_ = 4.681 (Figure 4) significantly (*P* < .05) changed the frequency of dopamine release events. Tukey post hoc analysis revealed that the 2.2 mg/kg heroin dose significantly increased the frequency of dopamine release events vs vehicle and the 0.27 mg/kg heroin (*P* < .001). In addition, the 1.1, 0.55, and 0.27 mg/kg heroin doses all increased the frequency of dopamine release events vs. vehicle (*P* < .001; Figure 3). By contrast, Tukey post hoc analysis revealed that only the second dose of buprenorphine (0.04 mg/kg IV) significantly increased dopamine release vs. vehicle (*P* < .001; Figure 4). Using the same data, we then performed a 2‐way repeated‐measures ANOVA to compare the effects of heroin on dopamine release when it is administered alone vs. when it administered after buprenorphine treatment (Figure 5). We found that buprenorphine pretreatment blocked the dopamine‐releasing effects of heroin. A two‐way repeated‐measures ANOVA revealed a significant interaction between treatment condition (heroin alone vs buprenorphine pretreatment) and heroin dose *F*_4,44_ = 5.71, *P* < .05. Tukey post hoc analysis revealed that, when compared to heroin‐alone animals, buprenorphine pretreatment significantly reduced the ability of heroin (1.1‐2.2 mg/kg IV) to increase the frequency of dopamine release events (Figure 5). Figure 6 illustrates working electrode lesion sites, thereby confirming that all recordings occurred in the NAc shell.

### 3.3 Pharmacokinetic analysis of plasma opioid content suggests that buprenorphine is metabolized into an active metabolite that may contribute to our dopamine observations

**FIGURE 7:**
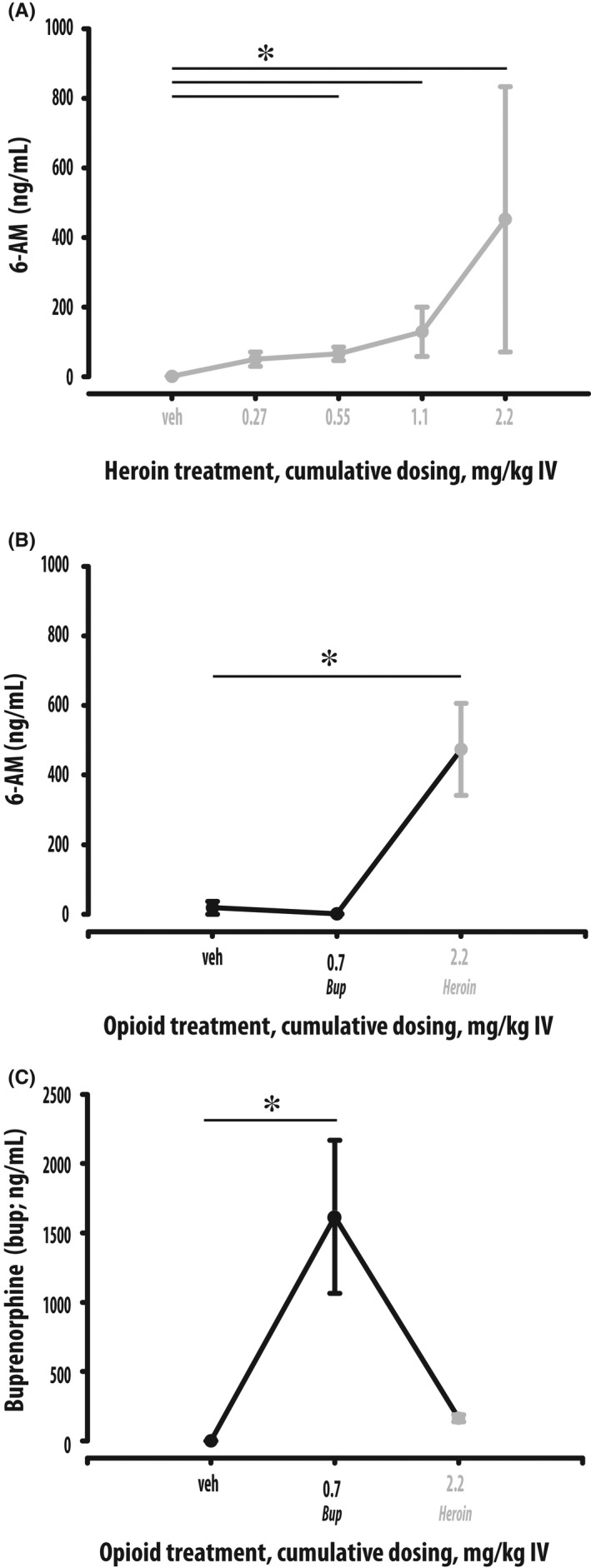
Pharmacokinetics of 6‐acetylmorphine (6‐AM) and buprenorphine (bup) in rats. A, Plasma levels of 6‐AM after each cumulative heroin injection when heroin is administered alone. The primary active metabolite of heroin, 6‐AM, lawfully increases with cumulative dose. B, Plasma levels of 6‐AM did not increase during buprenorphine cumulative dosing but did after heroin dosing. And, final 6‐AM concentration is comparable to those observed when heroin is administered without buprenorphine pretreatment (A). C, Plasma levels of buprenorphine increased after buprenorphine cumulative but returned to values that were comparable to vehicle by the final heroin injection. Horizontal bars indicate within‐group dose comparison. *P < .05

To measure just how much buprenorphine and 6‐acetylmorphine (6‐AM; active heroin metabolite) is in plasma at different time points in our cumulative‐dosing scheme, we performed an additional pharmacokinetic (PK) assay. We first treated naïve rats with the same cumulative doses of heroin used in our FSCV study (Figure 1A) and withdrew blood to analyze heroin plasma content every 10 minutes. We withdrew blood every 10 minutes to track concentration over the duration which we measured dopamine transients between cumulative treatments. Our data show that cumulative heroin dosing lawfully increases 6‐AM plasma concentration (Figure 7A). One‐way ANOVA on ranks revealed a significant main effect of heroin dose on 6‐AM levels (*H*_4_ = 18.54, *P* < .05). Tukey post hoc analysis further revealed that the 0.55, 1.1, and 2.2 mg/kg cumulative IV doses of heroin significantly increased 6‐AM versus vehicle. We next treated naïve rats (n = 6) with cumulative doses of buprenorphine followed by cumulative doses of heroin (Figure 1). We withdrew blood after vehicle and after each entire round of cumulative drug treatments. Our data show that cumulative dosing of buprenorphine followed by heroin resulted in a comparable increase in 6‐AM to that observed when heroin was administered by itself (Figure 7B). An unpaired t test between 6‐AM plasma values from heroin‐alone rats (Figure 7A) and 6‐AM plasma values from buprenorphine + heroin rats (Figure 7B) demonstrated that plasma levels were not statistically different between treatment groups 10 minutes following the final cumulative heroin injection (n.s.). Within the buprenorphine + heroin group, one‐way ANOVA on ranks revealed a significant main effect of heroin dose on 6‐AM levels (*H*_2_ = 11.47, *P* < .05). Tukey post hoc analysis further revealed that the 2.2 mg/kg cumulative IV dose of heroin significantly increased 6‐AM versus vehicle (*P* < .05). We also measured buprenorphine plasma levels in the buprenorphine + heroin group after each series of cumulative opioid treatments and found that buprenorphine plasma levels returned to baseline by the end of the final cumulative heroin treatment (ie, 40 minutes after last buprenorphine treatment; Figure 7C). One‐way ANOVA on ranks revealed a significant main effect of buprenorphine dose on buprenorphine plasma levels (*H*_2_ = 15.16, *P* < .05). Tukey post hoc analysis further revealed that the 0.7 mg/kg cumulative dose of buprenorphine significantly increased buprenorphine plasma content (*P* < .05) but buprenorphine plasma content was not distinguishable from vehicle 10 minutes following the final cumulative heroin treatment—a time point in which buprenorphine pretreatment prevented heroin‐evoked dopamine release.

## 4 DISCUSSION

In the present study, we used FSCV to measure how heroin, buprenorphine, and their combination affect transient dopamine release events in the NAc shell of freely moving rats. While these unique opioids both produced dose‐dependent changes in the frequency of dopamine release events, their effect on the amplitude of each event was less resolved. Analysis of the frequency data revealed that low‐dose buprenorphine functions as a weak dopamine releaser relative to heroin. When compared to vehicle, the frequency of dopamine release events maximally increased by ~25% following buprenorphine treatment and by ~60% following heroin treatment. However, distinct neurochemical effects emerged in the high‐dose range. While heroin lawfully increased the frequency of dopamine release events in a linear dose‐dependent manner, buprenorphine dosage produced a bell‐shaped effect on release. The frequency of buprenorphine‐evoked dopamine release events increased across the 0.01‐0.04 mg/kg range before declining to a level of insignificance across the 0.18‐0.7 mg/kg range. This latter observation confirms a previous microdialysis report demonstrating that buprenorphine alters accumbal dopamine concentration in a biphasic, dose‐dependent manner.^29^ One implication of these findings is that buprenorphine might only function as a dopamine‐based agonist therapy for opioid dependence in the low‐dose range. A bell‐shaped relationship between buprenorphine dose and dopamine release may also contribute to its relatively low IV abuse potential.

The opioid doses used in the present study were adapted from a double‐blind, placebo controlled inpatient trial. We selected these doses so that we could then compare our neurochemical findings to subjective responses that are impossible to measure in animal models. Similar to the present study, Comer and colleagues^18^ treated human subjects with ascending doses of either IV buprenorphine or heroin. In their study, both buprenorphine and heroin increased positive subjective reports; however, divergent responses were observed in the high‐dose range. Relative to high‐dose heroin (25 mg/70 kg in human; 2.24 mg/kg AED in rat), high‐dose buprenorphine (8 mg/70 kg in human; 0.7 mg/kg AED in rat) produced significantly fewer self‐reports of feeling good, feeling high, liking the drug, and desire to pay for drug.^18^ High‐dose buprenorphine was also unique because it was the only dose of any opioid tested to increase nausea and produce negative subjective reports among most (6 of 8) of the human participants.^18^ Because buprenorphine pharmacodynamics are more complex than heroin,^30^ it is likely that additional opioid receptor‐mediated effects occur in the high‐dose range. Mixed action at multiple opioid receptors may explain the bell‐shaped dose effect of buprenorphine on dopamine release and why high doses of buprenorphine produce negative subjective reports in human subjects.

Three classical opioid receptors (μ, δ, and κ) exist.^30^ Systemic administration of μ‐ and δ‐receptor agonists generally increases accumbal dopamine concentration; systemic administration of κ‐opioid receptor agonists generally decreases accumbal dopamine concentration.^30^ Heroin is a morphine derivative that is rapidly deacetylated into 6‐acetylmorphine in vivo. The antinociceptive and reinforcing effects of heroin are primarily attributed to 6‐acetylmorphine activating μ‐opioid receptor subtype1.^2^, ^31^ In addition to μ‐opioid receptor subtype 1, drug self‐administration studies suggest that δ‐, but not κ‐opioid receptors moderately influence heroin reinforcement.^2^ The pharmacodynamics of buprenorphine are more complex.^30^ Buprenorphine is known to bind to all major opioid receptors, although it displays a 10‐fold lower affinity for δ vs μ and κ. While buprenorphine is generally described as a partial μ‐opioid receptor agonist and a κ‐opioid receptor antagonist, it can act as a mixed agonist and/or antagonist at all major opioid receptor classes.^16^, ^17^, ^30^ In addition, buprenorphine acts at a recently identified opioid receptor known as the opioid‐like 1 receptor (ORL‐1R), which is thought to produce counter‐opioid effects.^30^ While our data do not provide definitive mechanistic insight into how buprenorphine biphasically alters dopamine release, we speculate that additional drug action at either the ORL‐1 and/or the κ‐opioid receptor may be involved because of their ability to produce counter‐opioid behavioral effects.

The complimentary PK analysis offers additional insight into the complex pharmacokinetic and pharmacodynamic mechanisms through which buprenorphine alters opioid‐evoked dopamine release. While the PK data confirm that our cumulative‐dosing scheme lawfully increased plasma levels of 6‐AM, we were surprised to find that buprenorphine was no longer detectable in plasma at a time point in which its pretreatment occluded heroin‐evoked dopamine release. This observation is supported by the findings of Ohtani et al^32^ who found the concentration of buprenorphine in brain tissue to be double that of the plasma 1‐hour post‐iv infusion. Thus, it is plausible that buprenorphine lingering postcumulative dosing is preventing heroin from binding to μ‐opioid receptors. Another potential explanation is that an active metabolite is involved and that the partial agonist buprenorphine is not simply functioning as a competitive μ‐opioid receptor antagonist in the presence of 6‐AM. Two well‐characterized active buprenorphine metabolites are buprenorphine‐3‐glucuronide and norbuprenorphine‐3‐glucuronide. Of these, it has been reported that buprenorphine‐3‐glucuronide exhibits high affinity for the μ‐ and δ‐ but not κ‐opioid receptors, whereas norbuprenorphine‐3‐glucuronide exhibits high affinity for κ‐ but not μ‐ or δ‐receptors (Brown et al^33^). Thus, one of several possibilities is that norbuprenorphine‐3‐glucuronide attenuates μ‐opioid‐induced increases in dopamine release by exerting additional action at the κ‐opioid receptor. A follow‐up study should be conducted to investigate whether κ‐opioid receptor antagonism blocks the effects of buprenorphine pretreatment on opioid‐evoked dopamine release. Another possibility is that buprenorphine is metabolized into an additional active metabolite that functions as a competitive antagonist at the μ‐receptor. Future studies are needed to identify if an active buprenorphine metabolite is blocking heroin‐evoked dopamine release, whether this effect results from competitive action at the μ‐, κ‐, and/or other receptor targets, or whether the effect is due to buprenorphine's longevity in brain tissue itself.

The μ‐opioid receptor‐mediated increase in accumbal dopamine concentration is theorized to arise from disinhibition of dopamine neurons in the VTA.^6^ Dopamine neurons are thought to be disinhibited when opioids activate μ‐opioid receptors on GABA neurons. The VTA is composed of not only dopamine (60%–65%), but also GABA (30%–35%) and glutamate (2%) neurons. Electrophysiological studies demonstrate that by activating μ‐opioid receptors on GABA neurons in the VTA, opioids increase accumbal dopamine release by disinhibiting tonically inhibited dopamine neurons.^6^ Recent optogenetics studies support that this pharmacodynamic mechanism underlies the reinforcing effects of heroin. Corre and colleagues^6^ found that animals will respond to transiently inhibit GABA neurons in the VTA but administering heroin occludes this effect. When considered in the context of the aforementioned electrophysiology literature, this finding supports the notion that opioids produce their reinforcing effects by activating μ‐opioid receptors on GABA neurons in the VTA, which then disinhibit dopamine neurons to increase accumbal dopamine concentration. It is likely that this disinhibitory mechanism contributes to the dopamine‐releasing effects of both buprenorphine and heroin.

While we attempted to make our dose assessments relevant for the clinical literature, caution should be used during the extrapolation of our dose‐dependent effects. Aside from being rats, our sample is different from that used by Comer et al^18^ in several ways. The opioid‐dependent subjects we attempted to model using drug naïve rats were not only dependent on opioids; they were also maintained with daily morphine treatment. Because repeated opioid exposure results in extensive μ‐opioid receptor desensitization throughout the brain,^34^, ^35^ it is likely that opioids are more potent and effective mediators of dopamine release in drug naïve rats vs. the dependent human subjects from the Comer sample. It should also be noted that the pharmacokinetics of sublingual (SL) Subutex^®^/Suboxone^®^ would produce distinct effects on dopamine release vs IV buprenorphine. Opioid bioavailability would also be significantly lower following SL vs IV buprenorphine. Future studies should be conducted to compare the interaction of buprenorphine and heroin on opioid‐evoked dopamine release in rats with a history of contingent drug self‐administration because the neurochemistry of acute noncontingent administration of drugs to animals is quite different than what occurs in models of reinforcement such as self‐administration. Thus, while it may be worth considering that Subutex^®^/Suboxone^®^ prescription guidelines suggest that physicians begin treatment with 8 mg/70 kg sublingual (SL) buprenorphine—a dose that is comparable to the highest buprenorphine dose used in the present study (0.7 mg/kg AED in rat), it is likely that IV buprenorphine is more potent and effective at increasing dopamine release vs. the same dose of SL buprenorphine.

Notwithstanding these limitations, our data provide new insight into how clinically relevant doses of heroin, buprenorphine, and their combination influence dopamine release. While heroin dose‐dependently increased the frequency of dopamine release events, buprenorphine only increased dopamine release in the low‐dose range. In addition, we found that buprenorphine pretreatment attenuates the dopamine‐releasing effects of heroin. Because high‐dose buprenorphine stopped increasing dopamine release and buprenorphine was no longer present in plasma, it is likely that its ability to blunt heroin‐evoked dopamine release is not exclusively a result of buprenorphine competing with 6‐AM at the μ‐opioid receptor.

## CONFLICT OF INTEREST

We have no conflicts to disclose.

## AUTHOR CONTRIBUTIONS

EBO designed the experiment and obtained funding to conduct it. DPI, RPL, TJE, HL‐B, and EBO performed the experiments and statistical analysis; DPI, LRH, and EBO wrote the manuscript. LRH and EBO finalized the manuscript. All authors read and approved the final manuscript.

## APPROVAL OF THE RESEARCH PROTOCOL BY AN INSTITUTIONAL REVIEWER BOARD

We need not require IRB review.

## INFORMED CONSENT

We did not require informed consent.

## REGISTRY AND THE REGISTRATION NO. OF THE STUDY/TRIAL

We did not register a clinical trial for this study.

## ANIMAL STUDIES

The University of Colorado Denver Institutional Animal Care and Use Committee approved all animal experiments and procedures in advance.

## Data Availability

The data which support these findings are available in the Oleson Lab GitLab^36^ page in the HeroinBuprenorphineVoltPK repository at [https://gitlab.com/oleson/heroinbuprenorphinevoltpk].
